# The putative tumor suppressor microRNA-30a-5p modulates clear cell renal cell carcinoma aggressiveness through repression of ZEB2

**DOI:** 10.1038/cddis.2017.252

**Published:** 2017-06-01

**Authors:** Zhenhua Chen, Jiaxing Zhang, Zhiling Zhang, Zihao Feng, Jinhuan Wei, Jun Lu, Yong Fang, Yanping Liang, Junjie Cen, Yihui Pan, Yong Huang, Fangjian Zhou, Wei Chen, Junhang Luo

**Affiliations:** 1 Department of Urology, The First Affiliated Hospital, Sun Yat-sen University, Guangzhou 510080, China; 2 Department of Oncology, The First Affiliated Hospital, Sun Yat-sen University, Guangzhou 510080, China; 3 Department of Urology, Sun Yat-sen University Cancer Center, Guangzhou 510080, China

## Abstract

Clear cell renal cell carcinoma (ccRCC), the most common subtype of renal cell carcinoma, can easily invade local tissues and metastasize, and is resistant to currently available treatments. Recent studies profiling microRNA expression in ccRCC have suggested miR-30a-5p may be deregulated in these cancer cells. To determine its role and mechanism of action in ccRCC, miR-30-5p expression levels were quantified and functions were analyzed using *in vitro* and *in vivo* experiments and bioinformatics. A decrease in miR-30a-5p expression was frequently noted in ccRCC cells and tissues. Importantly, low miR-30a-5p levels were significantly associated with a poor ccRCC patient prognosis. Stable overexpression of miR-30a-5p in 769-P cells was sufficient to prevent cellular proliferation and invasion *in vitro* and *in vivo*. Upon further examination, it was found that miR-30a-5p directly targeted the 3′-UTR of ZEB2 and suppressed ccRCC cell epithelial–mesenchymal transition. In addition, miR-30a-5p may be downregulated by the long non-coding RNA DLEU2. Taken together, these data reveal an important role for miR-30a-5p in the regulation of ccRCC proliferation and invasion, and indicate the potential for miR-30a-5p in applications furthering ccRCC prognostics and therapeutics.

MicroRNAs (miRNAs) are a class of conserved and small non-coding RNAs that downregulate protein expression by binding to complementary sequences present in the target mRNA.^1^ Recently, an increasing number of miRNAs have been reported to have functions in a diverse array of biological processes,^2, 3, 4^ including human cancer development and progression.^5, 6^

Renal cell carcinoma (RCC) accounts for ~5% of the malignancies in the United States.^7^ Of the types of RCC, clear cell renal cell carcinoma (ccRCC) is the most frequently occurring form (75–80%), can easily invade local tissue and metastasize and is resistant to currently available therapeutics.^8^ In addition, RCC patients respond poorly to radiation and conventional chemotherapy.^9^ Therefore, a more thorough understanding of the mechanisms underlying ccRCC pathogenesis will aid in developing more effective therapeutic strategies, for which there is an urgent need. Dysregulation of miRNAs has been observed in ccRCC in a number of different studies.^10, 11, 12, 13, 14^ Furthermore, several miRNAs, for example, miR-215, miR-200 s, miR-708, miR-205, miR-204, miR-199a and miR-141, have been reported to regulate ccRCC cell growth, apoptosis, migration and/or invasion,^13, 14, 15, 16, 17, 18, 19^ suggesting miRNA dysfunction may be associated with renal carcinogenesis. Obviously, more comprehensive research is required to illustrate the role of miRNAs during ccRCC progression and to identify miRNAs that could serve as novel prognostic predictors and therapeutic targets of ccRCC.

Previous studies profiling miRNAs have noted the downregulation of a number of miRNAs in ccRCC. One such downregulated miRNA identified was miR-30a-5p, which was one of the most influential in ccRCC pathogenesis.^20^ It has been reported that ectopic expression of miR-30a-5p in ccRCC cells inhibits cellular migration and invasion. In addition, miR-30a-5p has been demonstrated to suppress tumor growth in colon carcinomas,^21^ decrease cellular proliferation and invasion of Ewing tumors,^22^ inhibit cancer cell proliferation and induce apoptosis in liver cancers.^23^ Altogether, these data indicate a potential tumor suppressive function for miR-30a-5p. However, the role of miR-30a-5p in ccRCC and the molecular mechanisms by which it functions remain to be delineated.

In this present study, we report that downregulation of miR-30a-5p in ccRCC resulted in an aggressive phenotype and was correlated with a poor prognosis. Ectopic overexpression of miR-30a-5p in ccRCC cells was sufficient to inhibit cell invasion and metastasis both *in vitro* and *in vivo*. More importantly, this study provides the first evidence that miR-30a-5p directly targets the zinc-finger E-box binding homeobox 2 (ZEB2) mRNA. In addition, miR-30a-5p appeared to play a crucial role in regulating epithelial–mesenchymal transition (EMT) activity and thereby inhibited ccRCC cell invasiveness and metastasis. Furthermore, the long non-coding RNA (lncRNA) DLEU2 was demonstrated to reduce miR-30a-5p expression in ccRCC by acting as a miRNA sponge. Collectively, the results of this study provide an explanation behind the aggressiveness of ccRCC and link it mechanistically to interactions between miR-30a-5p, ZEB2 and DLEU2.

## Results

### Levels of miR-30a-5p are frequently lower in ccRCC cell lines and tissues, and low miR-30a-5p expression is associated with a poor ccRCC prognosis

**Figure 1:**
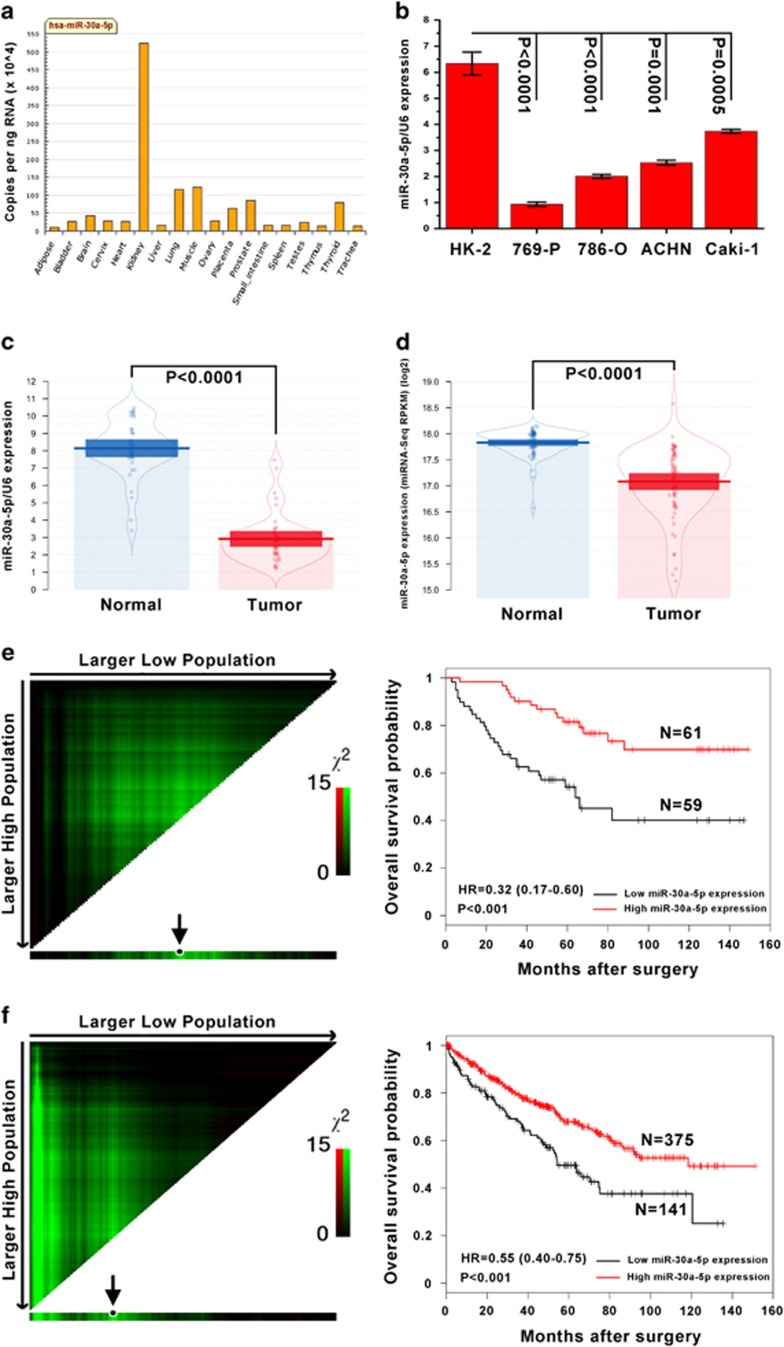
Mature miR-30a-5p expression levels in renal cell lines and tissues, and the prognostic value of miR-30a-5p levels in patients with ccRCC. (**a**) QPCR miR-30a-5p tissue expression specificity from miRNAMap database. (**b**) Expression levels of miR-30a-5p in HK-2 cell line and four ccRCC cell lines were examined by real-time PCR. Data are presented as mean±S.D. (*P*<0.001, independent *t*-test compared to HK-2 cell line). Experiments were performed three times. (**c**) Mature miR-30a-5p expression levels in 40 paired ccRCC and adjacent normal tissues obtained at the First Affiliated Hospital of Sun Yat-sen University. Alterations in expression are shown as pirateplot presentations, where the *y* axis indicates miR-30a-5p expression, and the points present raw data, the beans data density, the bars the mean of data and the band the 95% CI. The mean miR-30a-5p expression in ccRCCs was significantly lower than in normal tissues (*P*<0.0001, paired *t*-test). (**d**) Expression of mature miR-30a-5p in 68 paired ccRCC and adjacent normal tissues from TCGA database. The mean level of miR-30a-5p expression in the ccRCC tissues was significantly lower than in normal tissues (*P*<0.0001, paired *t*-test). (**e**) One hundred and twenty ccRCC patients from the First Affiliated Hospital and Cancer Center of Sun Yat-sen University, and (**f**) five hundred and sixteen ccRCC patients from the TCGA database. Left panel: X-tile plots automatically selected the optimum cutoff point. Plot coloration represents the strength of the association at each division, where red and green are inverse and direct associations, respectively, between marker expression and survival. The dark dots in the X-tile plots are indicated with an arrow and were used as cutoff points for dividing the patients into high- and low-expression groups. Right panel: Kaplan–Meier analysis of survival and the COX proportional hazards model for the hazard ratio of miR-30a-50p levels as a prognostic marker in ccRCC patients (*P*<0.001)

On the basis of a profile of miR-30a-5p tissue expression specificity, miR-30a-5p was found to be highly expressed in normal kidney tissue (Figure 1a). In this study, the expression levels of miR-30a-5p were examined by real-time PCR in five renal cell lines and 40 pairs of ccRCC, and adjacent normal tissues. All four ccRCC cell lines tested, 769-P, 786-O, ACHN and Caki-1, had lower levels of miR-30a-5p expression than the normal renal cell line HK-2 with the 769-P cell line having the least miR-30a-5p (Figure 1b). In primary ccRCC samples, miR-30a-5p levels were lower than in adjacent normal kidney tissue samples acquired from the First Affiliated Hospital and Cancer Center of Sun Yat-sen University (Figure 1c) and The Cancer Genome Atlas (TCGA) database (Figure 1d). To investigate the prognostic significance of miR-30a-5p levels in ccRCC patients, miR-30a-5p levels in 120 ccRCC tissues, including the 40 previously assessed samples, were measured using real-time PCR. The cutoff point for dividing tumors into low- and high-expression of miR-30a-5p was determined using X-tile software (Yale University School of Medicine, New Haven, CT, USA). Kaplan–Meier analysis revealed that low-level expression of miR-30a-5p was associated with an overall short survival time (*P*<0.001, HR=0.34 (0.18–0.64)) (Figure 1e). This prognostic value for miR-30a-5p expression levels was also confirmed using a large cohort of 516 ccRCC patients retrieved from the TCGA database (*P*<0.001, HR=0.55 (0.40–0.75)) (Figure 1f).

### MiR-30a-5p directly targets the 3′-UTR of ZEB2

**Figure 2:**
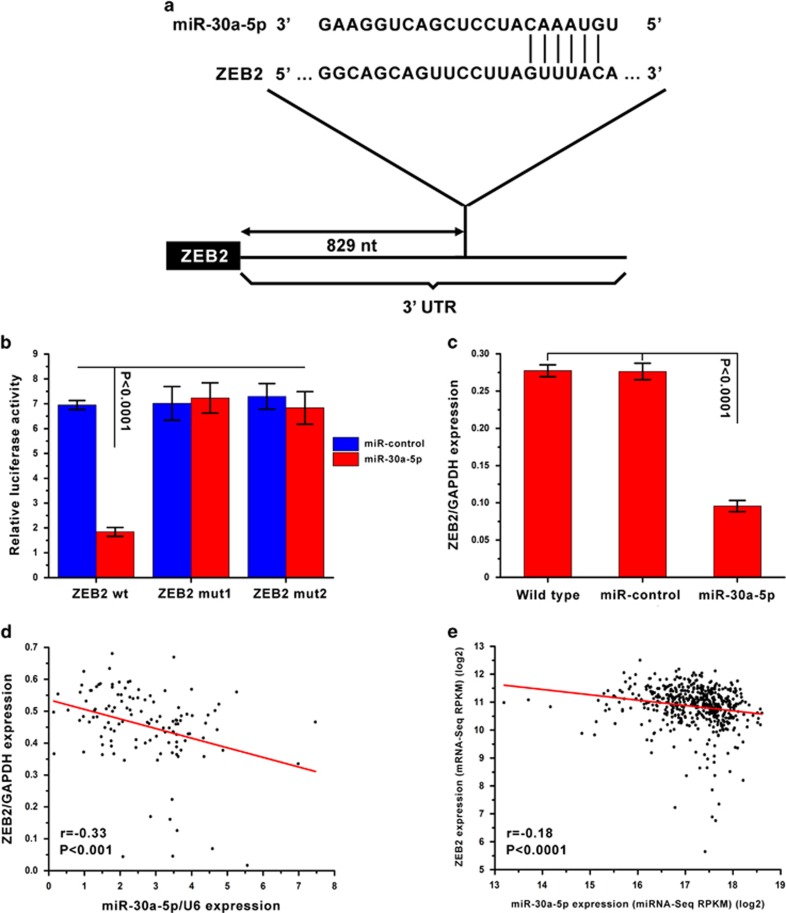
ZEB2 is the target of miR-30a-5p. (**a**) Schematic illustration of the predicted miR-30a-5p binding sites in the 3′-UTR of ZEB2. (**b**) ZEB2 was the target of miR-30a-5p. MiR report constructs containing one wild-type and two mutated ZEB2 3′-UTRs were co-transfected into 769-P cells, which were infected with either a miR-control-lentivirus or miR-30a-5p lentivirus. Relative repression of firefly luciferase expression was normalized to a transfection control. Results are presented as mean±S.D. and are representative of three independent experiments. (**c**) ZEB2 mRNA levels after transfection of miR-30a-5p into 769-P cells as measured by real-time PCR. (**d**) One hundred and twenty ccRCC patients from the First Affiliated Hospital and Cancer Center of Sun Yat-sen University, and (**e**) five hundred and fifteen ccRCC patients from the TCGA database. ZEB2 expression had an inverse correlation with miR-30a-5p expression in ccRCC patients

In general, miRNAs function by regulating expression of their downstream target gene(s). In this study, putative targets of miR-30a-5p were predicted using the target prediction databases miRWalk and microRNA.org. ZEB2 was identified as a potential target of miR-30a-5p, as the 3′-UTR of ZEB2 mRNA contains a site complementary to the seed region of miR-30a-5p (Figure 2a). To determine whether ZEB2 is a direct target of miR-30a-5p, the 3′-UTR of ZEB2, as well as two sequences with mutations in the miR-30a-5p binding site, were subcloned downstream of a luciferase reporter. These reporter constructs were then co-transfected into 769-P cells. It was found that luciferase activity was significantly affected by an increase in miR-30a-5p expression upon infection. By contrast, when the miR-30a-5p binding site was inactivated using site-directed mutagenesis, the luciferase activity of these mutant reporters was unaffected by the introduction of miR-30a-5p (Figure 2b). To verify miR-30a-5p affects ZEB2 expression in ccRCC intracellularly, ZEB2 expression was analyzed by real-time PCR in 769-P cells after miR-30a-5p overexpression and found to be markedly reduced compared to wild-type and miR-control cells (Figure 2c). Moreover, an inverse correlation between miR-30a-5p and ZEB2 expression levels was discovered in ccRCC tissues obtained from both the First Affiliated Hospital and Cancer Center of Sun Yat-sen University (*r*=−0.33, *P*<0.001) (Figure 2d) and the TCGA database (*r*=−0.18, *P*<0.0001) (Figure 2e). Collectively, these data support the bioinformatic prediction suggesting the 3′-UTR of ZEB2 is a direct target of miR-30a-5p.

### *In vitro* overexpression of miR-30a-5p suppresses ccRCC cell line growth, migration and invasion

**Figure 3:**
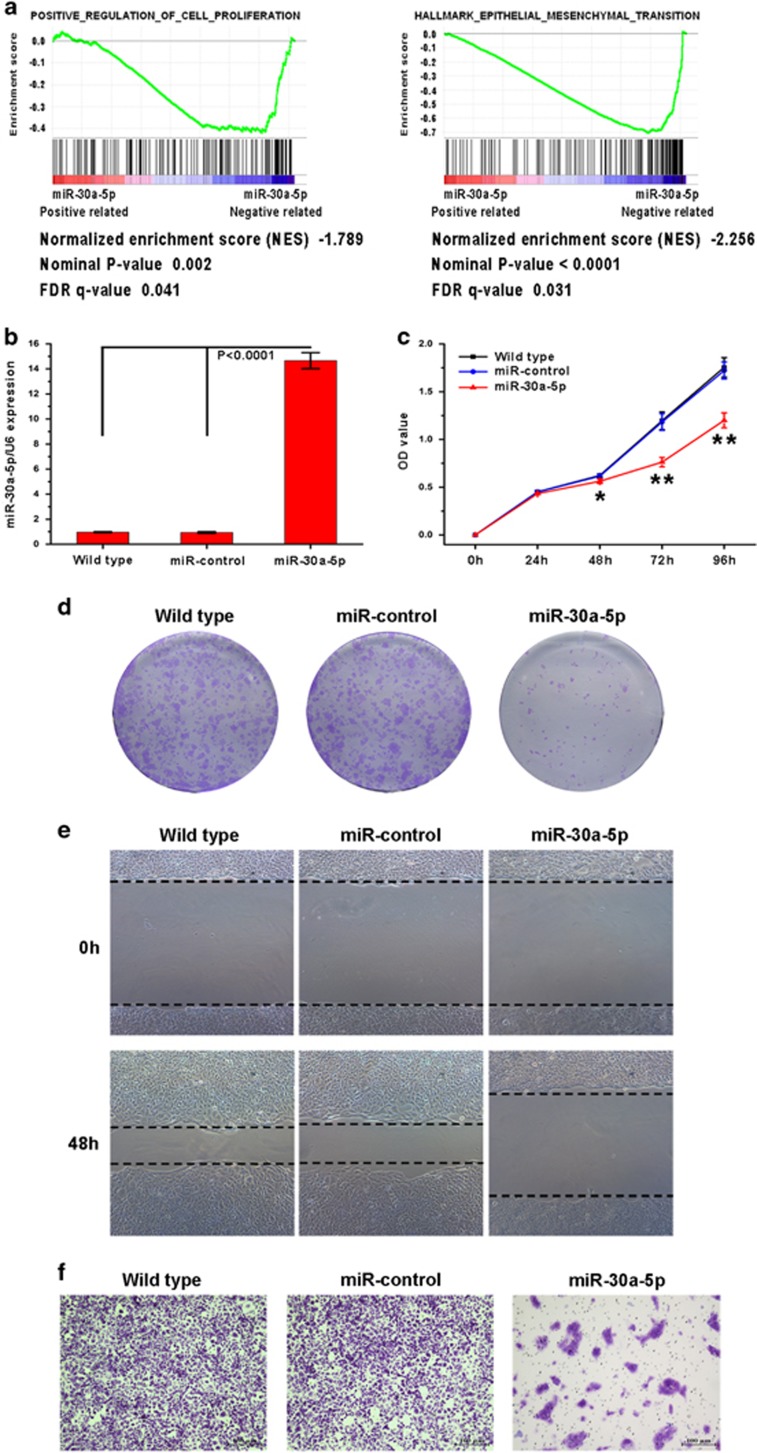
Exogenetic expression of miR-30a-5p suppresses ccRCC cell proliferation, colony formation, migration and invasion *in vitro*. (**a**) GSEA plots presenting the inverse correlation between miR-30a-5p expression with positive regulation of cell proliferation genes (left panel) and hallmark EMT genes (right panel) in the TCGA KIRC data set. (**b**) Real-time PCR analysis of miR-30a-5p in 769-P cells after exogenetic expression. (**c**) Cell growth of indicated cell as measured by CCK8 assay (*P<0.05 and **P<0.01 comparing exogenetic expression of miR-30a-5p and other treatments). (**d**) Effect of miR-30a-5p expression on ccRCC cell line colony formation. Representative colony formation results for wild-type, miR-control lentivirus-infected and miR-30a-5p lentivirus-infected 769-P cells, where the results were independently reproducible in triplicate. (**e**) Wound healing assay assessing cell motility in wild-type 769-P, miR-control 769-P and miR-30a-5p 769-P cells. Overexpression of miR-30a-5p notably inhibited the migration of 769-P cells. (**f**) Cell invasion was evaluated using the Matrigel invasion chamber, and miR-30a-5p overexpression clearly inhibited 769-P cell invasion

Next, the biological functions of miR-30a-5p in ccRCC tumorigenesis and progression were explored. As expected, gene set enrichment analysis (GSEA) revealed that miR-30a-5p expression was inversely correlated with the expression of two gene sets previously defined in the TCGA KIRC (kidney renal clear cell carcinoma) data set, where one is of genes involved in positive regulation of cell proliferation and the other is hallmark genes of EMT (Figure 3a). Upon transfection, miR-30a-5p expression increased in the 769-P cell line as measured by real-time PCR (Figure 3b). Next, the ability of 769-P cells to proliferate when left uninfected or after infection with miR-30a-5p or the miR-control was evaluated. The CCK8 assay revealed that miR-30a-5p overexpression significantly decreased cell viability (Figure 3c). In colony formation assay, miR-30a-5p-transfected cells created notably fewer and smaller colonies than the miR-control-transfected and wild-type cells (Figure 3d), indicating a role for miR-30a-5p in inhibiting growth of ccRCC cells.

The effect of miR-30a-5p on the ability of ccRCC cells to migrate and invade was then characterized using wound healing and Matrigel invasion assays. Overexpression of miR-30a-5p was found to suppress 769-P cell migration in the wound healing assay (Figure 3e) and invasion in the Matrigel assay (Figure 3f).

### Exogenetic overexpression of miR-30a-5p inhibits proliferation and metastasis of ccRCC cells *in vivo*

**Figure 4:**
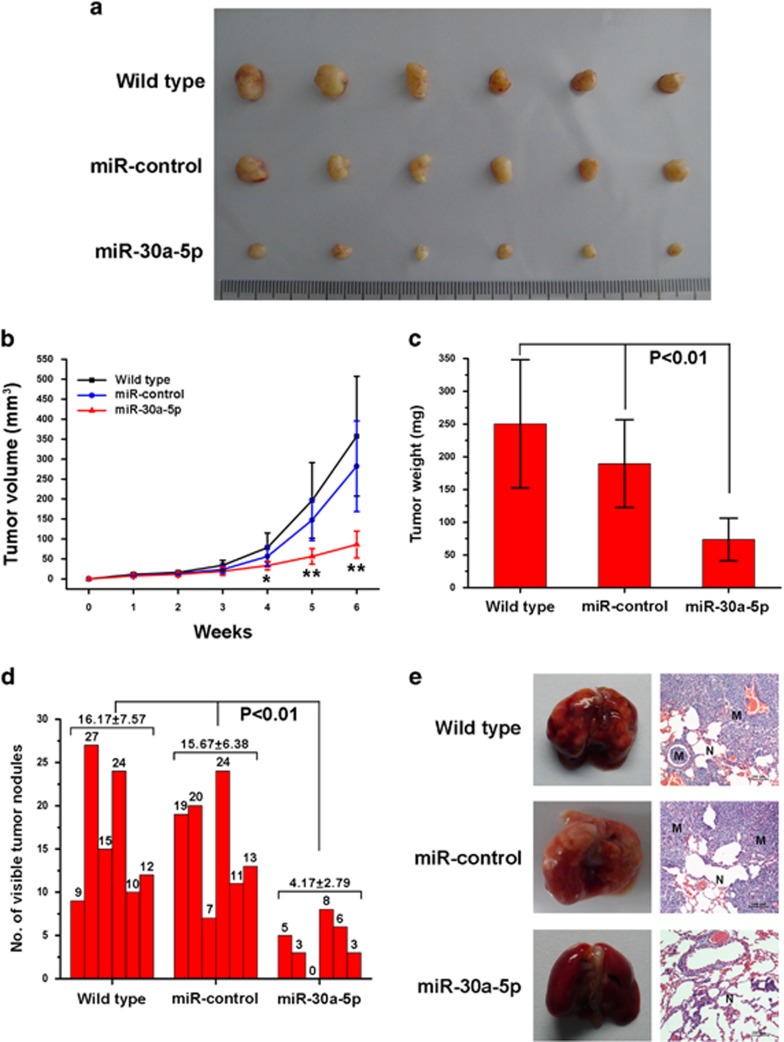
Exogenetic expression of miR-30a-5p reduces ccRCC cell line proliferation and metastasis *in vivo*. (**a**) Actual sizes of representative tumors. (**b**) Mean tumor volume (±S.D.) (*n*=6, *P<0.05 and **P<0.01 comparing exogenetic expression of miR-30a-5p and other treatments). Tumor diameter was measured using a digital caliper and tumor volume was calculated twice a week using the formula volume=*W*^2^ × *L*/2. (**c**) Mean tumor weight (±S.D.) (*n*=6, *P*<0.01 comparing exogenetic expression of miR-30a-5p and other treatments). (**d**) The number of nodules were quantified in the lungs of athymic mice 6 weeks after tail vein injection of wild-type, miR-control lentivirus-infected and miR-30a-5p lentivirus-infected 769-P cells (*n*=6 per group). The nodules were examined under an anatomical microscope (*P*<0.01, Mann–Whitney *U*-test) (**e**) Left panel: representative metastatic nodules on the surface of the lungs of athymic mice. Right panel: H&E staining of a section of metastatic tumors in the lung (M) and normal lung (N). Original magnification × 100

The function of miR-30a-5p in ccRCC has never been studied *in vivo*. Therefore, to investigate the effect of miR-30a-5p on cancer cell proliferation, wild-type, miR-control-transfected and miR-30a-5p-transfected 769-P cells were introduced into a nude mouse xenograft model. Forty-two days post injection of the cells, the volume and weight of tumors in mice injected with cells transfected with miR-30a-5p were markedly lower than in the other cohorts (*P*<0.01 in tumor volume and weight comparisons) (Figures 4a–c). Meanwhile, no significant differences were observed in tumor volume or weight between the wild-type and miR-control groups. To study the effect of miR-30a-5p overexpression on metastasis *in vivo*, an experimental metastasis model was used, where wild-type, miR-control-transfected and miR-30a-5p-transfected 769-P cells were injected into the lateral tail vein of athymic nude mice (six mice per group). As shown in Figure 4d, the mice injected with the miR-30a-5p overexpressing 769-P cells had fewer nodes per lung than the mice in the other groups (*P*<0.01). Histological studies identified that the nodules were caused by extravasation of 769-P cells into the lungs and subsequent tumor growth (Figure 4e).

### Overexpression of miR-30a-5p suppresses ccRCC cell EMT, migration and invasiveness by targeting ZEB2

**Figure 5:**
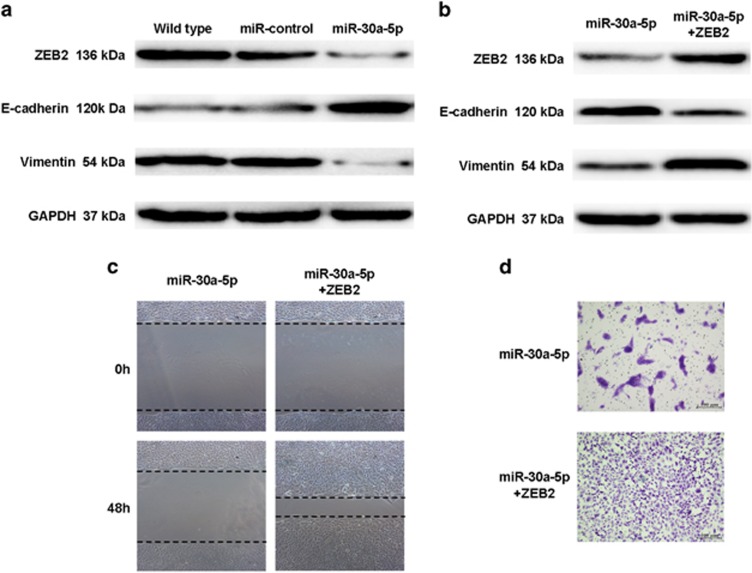
*In vitro* overexpression of miR-30a-5p in 769-P cells reverses EMT through regulation of ZEB2. (**a**) Expression of epithelial and mesenchymal markers was measured by western blot in wild-type, miR-control and miR-30a-5p 769-P cells. GAPDH was used as a loading control. (**b**) ZEB2 was transfected into miR-30a-5p 769-P cells. Western blot demonstrating that miR-30a-5p inhibition of EMT was abrogated in miR-30a-5p 769-P cells upon ectopic expression of ZEB2. (**c**) Wound healing and (**d**) invasion assays showing ectopic expression of ZEB2 in miR-30a-5p 769-P cells abrogated miR-30a-5p suppression of migration and invasion of 769-P cells

ZEB2 has been implicated in EMT induction, where it acts by directly repressing E-cadherin and indirectly promoting Vimentin expression.^24, 25^ In a previous study, we also reported a correlation between ZEB2 and EMT markers expression.^26^ Since it is well-known that EMT is involved in invasion and metastasis of cancer cells, it was tested whether miR-30a-5p levels in ccRCC cells affect the induction of EMT. To this end, epithelial and mesenchymal marker expression was measured by western blot in wild-type, miR-control and miR-30a-5p overexpressing 769-P cells. Upon miR-30a-5p overexpression, it was found E-cadherin expression increased, while the mesenchymal marker Vimentin decreased in 769-P cells (Figure 5a). It was then examined if ZEB2 was capable of counteracting the miR-30a-5p-mediated suppression of EMT. Stable miR-30a-5p expressing 769-P cells were transfected with pcDNA3.1 (+)-ZEB2, which contained the entire ZEB2 coding sequence except for the 3′-UTR. As expected, ZEB2 overexpression in miR-30a-5p 769-P cells nullified the miR-30a-5p reversal of EMT (Figure 5b). Moreover, miR-30a-5p suppression of migration and invasion was abrogated (Figures 5c and d). Taken together, these results provide evidence that ZEB2 is involved in miR-30a-5p regulation of ccRCC cell migration and invasion.

### The long non-coding RNA DLEU2 reduces miR-30a-5p expression in ccRCC cells

**Figure 6:**
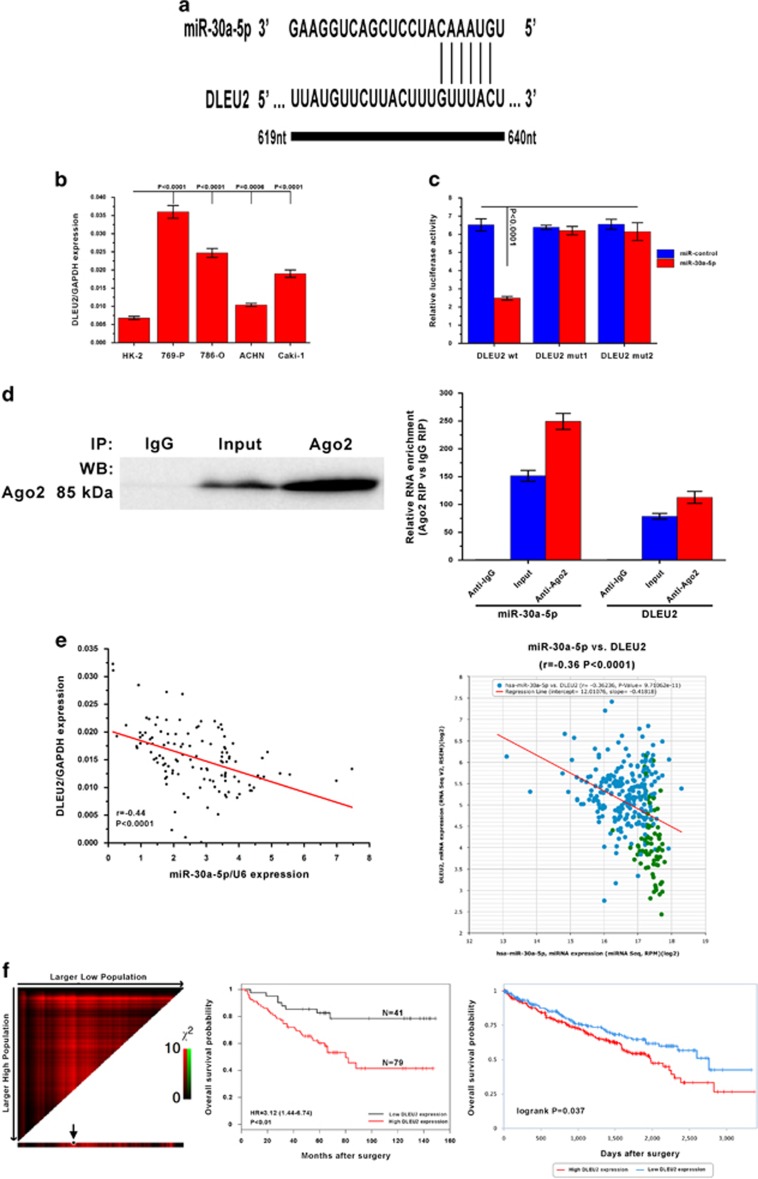
DLEU2 reduces miR-30a-5p expression in ccRCC. (**a**) Putative DLEU2 RNA binding sequence in miR-30a-5p. (**b**) Expression levels of DLEU2 in HK-2 cell line and four ccRCC cell lines were examined by real-time PCR. Data are presented as mean±S.D. (*P*<0.001, independent *t*-test compared to HK-2 cell line). Experiments were performed three times. (**c**) MiR report constructs that included one wild-type and two mutant DLEU2 binding sequences were co-transfected into 769-P cells infected with miR-control-lentivirus or miR-30a-5p lentivirus. Relative repression of firefly luciferase expression was normalized against a transfection control. Results are presented as mean±S.D. for three independent experiments. (**d**) Lysates of 769-P cells underwent RIP with the Ago2 antibody. Left panel: Ago2 was detected using IP-western (left panel), and DLEU2 and miR-30a-5p using real-time PCR. RNA levels are presented as fold enrichment of Ago2 relative to IgG immunoprecipitates (right panel). (**e**) DLEU2 expression correlated with miR-30a-5p expression in ccRCC samples from the First Affiliated Hospital and Cancer Center of Sun Yat-sen University (left panel), and the TCGA data set from starBASE v2.0 (right panel). (**f**) DLEU2 expression was inversely associated with survival of ccRCC patients from the First Affiliated Hospital and Cancer Center of Sun Yat-sen University (left panel: X-tile plot and middle panel: Kaplan–Meier analysis for survival and COX proportional hazards model for hazard ratio) and the TCGA data set from TANRIC database (right panel: Kaplan–Meier analysis for survival)

Increasing evidence has shown that lncRNAs contain motifs with sequences complementary to miRNAs and can inhibit miRNAs expression and activity.^27, 28^ To examine whether miR-30a-5p is regulated in such a manner in ccRCC cells, interactions between miR-30a-5p and lncRNAs were predicted using starBASE v2.0. The lncRNA DLEU2 was identified as containing a conserved target site in the miR-30a-5p seed region (Figure 6a). Then DLEU2 expression levels in all four ccRCC cell lines, 769-P, 786-O, ACHN and Caki-1, and the normal renal cell line HK-2 were tested. The result showed an inverse correlation between DLEU2 and miR-30a-5p expression in most cell lines (Figure 6b). Therefore, wild-type and mutant DLEU2 sequence constructs were subcloned into the pMIR luciferase reporter and then co-transfected into 769-P cells with miR-30a-5p or the miR-control. The luciferase activity of cells pMIR-DLEU2 was significantly decreased upon co-transfection with miR-30a-5p, while the luciferase activity in cells in the other treatment cohorts was unaffected (Figure 6c). It is well-accepted that miRNAs regulate their targets through formation of RNA-induced silencing complex (RISC). Moreover, lncRNAs can regulate miRNA activity by acting as molecular sponges by associating with RISC.^29, 30^ To investigate whether DLEU2 and miR-30a-5p are part of a RISC complex, RNA-binding protein immunoprecipitation (RIP) experiment was conducted on 769-P cells lysates using an antibody against Ago2, a key component of the RISC complex. It was confirmed that the Ago2 antibody successfully precipitated the Ago2 protein from cellular extracts (Figure 6d, left panel). When DLEU2 RNA and miR-30a-5p levels were quantified in the immunoprecipitates by qRT-PCR, they were found to be enriched in Ago2 immunoprecipitates compared to control IgG immunoprecipitates (Figure 6d, right panel). Overall, in accordance with the bioinformatics analysis and luciferase assay, these results suggest DLEU2 is present in Ago2-containing RISC associated with miR-30a-5p. Furthermore, when miR-30a-5p and DLEU2 expression was measured, a correlation was found between miR-30a-5p and DLEU2 expression in tissues from patients in the ccRCC patient cohort (*r*=−0.44, *P*<0.0001) and TCGA database (*r*=−0.36, *P*<0.0001) (Figure 6e). As stated above, miR-30a-5p expression is associated with a favorable prognosis in ccRCC patients. Therefore, it was determined if there was a prognostic role for DLEU2 expression levels in ccRCC patients. As shown in Figure 6f, higher expression of DLEU2 was associated with a poor prognosis in ccRCC patients from both the patient cohort and TCGA database (*P*<0.05), supporting the inverse correlation between miR-30a-5p and DLEU2 expression.

## Discussion

While miRNA dysregulation has been described in several types of human cancers,^31, 32^ the underlying mechanisms by which miRNAs regulate carcinogenesis remain unclear. In this study, downregulation of miR-30a-5p was a frequent occurrence in ccRCC tissues, and low miR-30a-5p expression had a significant association with poor ccRCC patient survival. In functional studies, proliferation, colony formation, migration and invasion of ccRCC cells *in vitro*, and tumor growth and metastasis *in vivo* were dramatically suppressed upon reintroduction of miR-30a-5p. These results suggest that miR-30a-5p plays a crucial role in the growth, invasiveness and metastatic potential of ccRCC.

Invasion and metastasis are two of the most important hallmarks of malignant tumors and are factors directly correlated with mortality in human cancers. Therefore, it is essential to identify factors involved in the ability of tumor cells to invade and metastasize, as well as explore the underlying molecular mechanisms involved in the progression of tumor metastasis. Furthermore, EMT, which facilitates cell motility and invasion, is a key event in tumor invasion and metastasis.^33, 34^ Therefore, it was determined whether ZEB2 is a potential functional target of miR-30a-5p, as ZEB2 has been shown to have an important role in the EMT. In this study, it was shown that miR-30a-5p binds to a complementary site, which is conserved among most vertebrates on the 3′-UTR of ZEB2, resulting in a marked decrease in ZEB2 expression. In addition, a significant inverse correlation was identified between miR-30a-5p and ZEB2 mRNA levels in both the ccRCC patient and TCGA KIRC cohorts. To the authors’ knowledge, these observations provide the first evidence that miR-30a-5p mechanistically acts through the regulation of ZEB2. It has been reported that ZEB2 is upregulated in several types of human cancers, including ccRCC, and overexpression of this protein is positively correlated with tumor metastasis and poor prognosis.^26, 35, 36, 37, 38^ This is consistent with our findings that miR-30a-5p downregulation is associated with a poor ccRCC prognostic phenotype. Moreover, as mentioned above, ZEB2 can induce EMT by directly repressing E-cadherin and indirectly promoting Vimentin. In ccRCC cells, it appears miR-30a-5p modulates tumor cell migration and invasion through regulation of ZEB2. As shown by the enhanced expression of the epithelial marker E-cadherin and decreased expression of the mesenchymal marker Vimentin, the reintroduction of miR-30a-5p into 769-P cells reversed EMT by acting through ZEB2. Furthermore, the exogenous introduction of ZEB2 caused a substantial recovery in EMT, migration and invasiveness in miR-30a-5p expressing 769-P cells. These results support ZEB2 acting as the predominant mediator of miR-30a-5p inhibition of invasiveness of ccRCC cells, suggesting a loss in miR-30a-5p function may result in an enhanced expression of ZEB2 that promotes cellular invasion and metastasis.

In addition, it was found DLEU2, a lncRNA regarded as a tumor suppressor in leukemia,^39^ can downregulate miR-30-5p expression in ccRCC cells by acting as a miRNA sponge. The distinct functions of DLEU2 in different tumors may be a result of the different DLEU2 transcripts generated by alternative splicing. A DLEU2-induced reduction in miR-30a-5p may result in a derepression of the mRNA targets of miR-30a-5p, such as ZEB2, and facilitate the malignant progression of ccRCC cells through a competing endogenous RNA mechanism. Thus, interactions between DLEU2, miR-30a-5p and ZEB2 may be biologically significant in the network regulating ccRCC tumor aggressiveness.

In summary, we investigated the potential functions and mechanisms of action of miR-30a-5p in ccRCC proliferation and aggressiveness. The results of this study suggest that downregulation of miR-30a-5p plays an important role in ccRCC cell growth and metastasis. In addition, miR-30a-5p expression may be regulated by lncRNA DLEU2. However, the detailed mechanism behind miR-30a-5p and DLEU2 interactions, and other means by which miR-30a-5p is downregulated, such as through DNA promoter methylation, still need to be elucidated in future studies. Importantly, miR-30a-5p has potential as a new prognostic marker for and/or an effective therapeutic target against ccRCC.

## Materials and Methods

### Tissue samples and cell culture

ccRCC and adjacent normal tissue samples were collected from patients between 2002 and 2010 at the First Affiliated Hospital and Cancer Center of Sun Yat-sen University in Guangzhou, China. Informed consent was obtained under institutional review board-approved protocols. Two ccRCC cell lines, 769-P and 780-O, were cultured in RPMI-1640 medium with 10% fetal bovine serum (FBS). Another two ccRCC cell lines, ACHN and Caki-1, were maintained in MEM–EBSS medium with 10% FBS and McCoy’s 5 A medium with 10% FBS, respectively. Normal renal cell line HK-2 was in DMEM/F12 with 10% FBS.

### Bioinformatics analysis databases

The expression profile of miR-30a-5p in different tissues was obtained from the miRNAMap database.^40^ Putative miR-30a-5p targets were predicted using miRWalk 2.0 and the microRNA.org database.^41, 42^ The KIRC patient clinical and RNA-Seq data were from TCGA and downloaded from the Broad GDAC Firehose database (Broad Institute TCGA Genome Data Analysis Center (2016): Analysis-ready standardized TCGA data from Broad GDAC Firehose 2016_01_28 run. Broad Institute of MIT and Harvard. Data set. http://doi.org/10.7908/C11G0KM9). Interactions between miRNA and lncRNA were assessed using starBASE v2.0 and the lncRNA Kaplan–Meier survival analysis was conducted with the TANRIC database.^43, 44^

### Gene set enrichment analysis

Global mRNA expression profiles within the TCGA KIRC subset for which miR-30a-5p expression data were available were imported into GSEA to identify associations of miR-30a-5p using the Molecular Signatures Database (MSigDB.v5.1.symbols). For GSEA, miR-30a-5p expression was regarded as a numeric variable. A continuous-type cls file of the miR-30a-5p profile was applied to phenotype labels in GSEA. The metric for ranking genes in GSEA was set as ‘Pearson’, the plot graphs for the top sets for each phenotype were set to 150 and the other parameters were set to default values. GSEA was performed using GSEA v2.2.0 software (Cambridge, MA, USA).

### RNA isolation and quantitative real-time PCR

Total RNA was extracted using TRIzol (Invitrogen, Carlsbad, CA, USA) and miRNA cDNA was synthesized using the Mir-X miRNA First Strand Synthesis Kit (Clontech, Mountain View, CA, USA), and mRNA and lncRNA cDNA was synthesized using the PrimeScript RT reagent Kit (Promega, Madison, WI, USA). Real-time PCR was carried out on a Bio-Rad CFX96 real-time PCR system (Bio-Rad, Hercules, CA, USA). The sequences of the primers used were as follows:

miR-30a-5p primers:

F: 5′-TGTAAACATCCTCGACTGGAAG-3′

R: mRQ 3′ primer provided in the Mir-X miRNA First Strand Synthesis Kit

U6 primers:

F: 5′-ACGCAAATTCGTGAAGCGTT-3′

R: mRQ 3′ primer provided in the Mir-X miRNA First Strand Synthesis Kit

ZEB2 primers:

F: 5′-CAAGAGGCGCAAACAAGCC-3′

R: 5′-GGTTGGCAATACCGTCATCC-3′

DLEU2 primers:

F: 5′-TCCGAGAGTATAGCGCCACT-3′

R: 5′-ACTGCCCTTTGCTCCAAGTA-3′

GAPDH primers:

F: 5′-CCCACATGGCCTCCAAGGAGTA-3′

R: 5′-GTGTACATGGCAACTGTGAGGAGG-3′

### Vector construction

The pre-miR-30a-5p and ZEB2 coding sequences were amplified and cloned into pCDH-CMV-MCS-EF1-coGFP (System Biosciences, Mountain View, CA, USA) and pcDNA3.1 (+) to generate pCDH-CMV-miR-30a-5p and ZEB2 expression vectors, respectively. The primer sequences used were as follows:

Pre-miR-30a-5p primers:

F: 5′-GCATCTCGAGGCTGTTTGAATGAGGCTTCA-3′

R: 5′-GCATCTCGAGCCATTTTAATTCAGCTTTGT-3′

ZEB2 CDS primers:

F: 5′-CGATATCATGAAGCAGCCGATCATG-3′

R: 5′-CGTCGACTTACATGCCATCTTCCATATTGT-3′

### Lentivirus production and transduction

Viral particles were harvested 48 h after transfection of 293FT cells with pCDH-CMV-miR-30a-5p and the packaging plasmids pRSV/pREV, pCMV/pVSVG and pMDLG/pRRE using Lipofectamine 2000 (Invitrogen). Recombinant lentivirus-transducing units were used to infect 769-P cells in the presence of 8 mg/ml Polybrene (Sigma, St Louis, MO, USA).

### Luciferase reporter assay

The putative miR-30a-5p binding site on the 3′-UTR of ZEB2 mRNA and the binding sequence of DLEU2 binding sequence were independently cloned downstream of the cytomegalovirus (CMV) promoter in a pMIR-REPORT vector (Ambion, Carlsbad, CA, USA). Two mutant constructs were generated by either deletion or mutation. The firefly luciferase construct and control Renilla luciferase vector were co-transfected into 769-P cells with either miR-30a-5p or miR-control. A dual luciferase assay (Promega) was performed 48 h post transfection. Experiments were performed in triplicate. The primer sequences used are as follows:

miR-30a-5p-ZEB2 primers:

F: 5′-AAAAAACTAGTTAAACTACTGCATTTTAAGCTTC-3′

R: 5′-AAAAAGTTTAAACAGTTTGGCTACATTTTTATTCGA-3′

miR-30a-5p-ZEB2 mut1 primers:

F: 5′-AAAAAACTAGTTATGTTTGTGCAATTATTTTCTGTA-3′

R: 5′-AAAAAGTTTAAACTTGTATTTAACAGTCCCTCT-3′

miR-30a-5p-ZEB2 mut2 primers:

F: 5′-AAAAAACTAGTCAGCAGTTCCTTAGGTTCCATATGTTTGTGC-3′

R: 5′-AAAAAGTTTAAACCATTGTATTTAACAGTCCCT-3′

miR-30a-5p-DLEU2 primers:

F: 5′-AAAAAACTAGTCGCCATTTTCGAGTGATGCC-3′

R: 5′-AAAAAGTTTAAACATTTCATATAGGCTTAGAAAAAAAA-3′

miR-30a-5p-DLEU2 mut1 primers:

F: 5′-AAAAAACTAGTACTTGGAGCAAAGGGCAGTC-3′

R: 5′-AAAAAGTTTAAACAAAGTAAGAACATAAACTTAA-3′

miR-30a-5p-DLEU2 mut2 primers:

F: 5′-AAAAAACTAGTATGTTCTTACTTTGGTTCCTTATAA-3′

R: 5′-AAAAAGTTTAAACAGTCCATAAAGCCTACAGAA-3′

### CCK8 assay for cell growth and viability

In the wells of a 96-well plate, 5 × 10^3^ cells were seeded and incubated for 24, 48, 72 or 96 h. Cell growth and viability was measured using a Cell Counting Kit-8 (Beyotime, Shanghai, China) according to the manufacturer’s instructions. Absorbance was measured at 450 nm in an Elx800 Reader (Bio-Tek Instruments Inc., Winooski, VT, USA).

### Colony formation assay

Twenty-four hours post infection, 500 untreated or infected cells were placed into separate wells of a six-well plate and cultured in RPMI-1640 with 10% FBS for 2 weeks. Colonies were fixed with methanol and stained with 0.1% crystal violet in 20% methanol for 15 min.

### Wound healing and invasion assays

An area covered in cells was scratched with a 200 ml pipette, and wound closure was observed and photographed under a microscope after 48 h. For invasion assays, 10^5^ cells were added to a Matrigel invasion chamber (BD Biosciences, Becton Dickson Labware, Flanklin Lakes, NJ, USA) in a 24-well culture plate and FBS was added to the lower chamber. After 48 h, the non-invading cells were gently removed with a cotton swab. Invasive cells, which were located in the lower chamber, were stained with crystal violet, air-dried and photographed.

### Subcutaneous xenograft and lung metastasis model

Female BALB/c-nu/nu athymic mice (4–5 weeks old) were purchased from Shanghai Slac Laboratory Animal Co. Ltd. (Shanghai, China). Animal care and experimental protocols used in this study were approved by the Institute Research Medical Ethics Committee of Sun Yat-sen University.

For the subcutaneous xenograft model, three groups of six mice each were injected subcutaneously at the same site with 10^6^ prepared cells. Tumor volume was calculated using the formula, *V*=*W*^2^ × *L*/2, where *V* refers to volume, *W* refers to short axial length and *L* refers to long axial length. Animals were killed 42 days post injection and their tumors were weighed. For the lung metastasis model, 2 × 10^5^ prepared cells were injected intravenously through the tail vein into each mouse in a laminar flow cabinet. Six weeks post injection, the mice were killed and examined.

### Western blot

Equal amounts of whole-cell lysates were resolved by electrophoresis on a sodium dodecylsulfate polyacrylamide gel and transferred onto a polyvinylidene difluoride membrane (Pall Corp., Port Washington, NY, USA). The membrane was incubated with primary antibodies overnight at 4 °C, washed three times with TBST, and then incubated with secondary antibodies at room temperature for 1 h. Primary and secondary antibodies were diluted in 5% milk in TBST. Proteins recognized by these antibodies were visualized with enhanced chemiluminescence detection reagents (Amersham Biosciences, Uppsala, Sweden) according to the manufacturer’s instructions. Rabbit anti-ZEB2, anti-E-cadherin and anti-Vimentin antibodies, and mouse anti-GAPDH antibodies were from Abcam (Cambridge, UK).

### RNA immunoprecipitation assay

To perform RIP, the Magna RIP RNA-Binding Protein Immunoprecipitation Kit (Millipore, Billerica, MA, USA) and the Ago2 antibody (Millipore) were used according to the manufacturer’s protocol. Briefly, cells were lysed in RIP lysis buffer, and then 100 *μ*l was incubated with RIP buffer containing A+G magnetic beads conjugated with human anti-Ago2 antibody, where normal mouse IgG (Millipore) served as a negative control and anti-snRNP70 as a positive control (Millipore). Samples were incubated with Proteinase K with shaking to digest the protein and then RNA was immunoprecipitated and isolated. Finally, qRT-PCR was performed to detect miR-30a-5p and DLEU2 in the precipitates.

### Statistical analysis

Data are represented as mean±S.D. Assessment of statistically significant differences in continuous and discrete data was performed using Student’s two-tailed *t*-tests and Wilcox tests, respectively. Correlations were analyzed by Pearson’s correlation test, the two patient cohorts were compared using a Kaplan–Meier survival plot, and the Cox proportional hazards model was used to compute the hazard ratio. *P*<0.05 was considered statistically significant. X-tile software version 3.6.1 with a built-in validated feature that automatically defined the cutoff point.^45^ For statistical assessments and plotting, R software version 3.3.1 (R Foundation for Statistical Computing, Vienna, Austria) and OriginPro 9 (OriginLab, Northampton, MA, USA) were used.
